# TRPM2 Channel in Microglia as a New Player in Neuroinflammation Associated With a Spectrum of Central Nervous System Pathologies

**DOI:** 10.3389/fphar.2019.00239

**Published:** 2019-03-12

**Authors:** Philippa Malko, Sharifah A. Syed Mortadza, Joseph McWilliam, Lin-Hua Jiang

**Affiliations:** ^1^School of Biomedical Sciences, Faculty of Biological Sciences, University of Leeds, Leeds, United Kingdom; ^2^Department of Biochemistry, Universiti Putra Malaysia, Seri Kembangan, Malaysia

**Keywords:** TRPM2 channel, microglial cell activation, CNS pathologies, neuroinflammation, proinflammatory mediators

## Abstract

Microglial cells in the central nervous system (CNS) are crucial in maintaining a healthy environment for neurons to function properly. However, aberrant microglial cell activation can lead to excessive generation of neurotoxic proinflammatory mediators and neuroinflammation, which represents a contributing factor in a wide spectrum of CNS pathologies, including ischemic stroke, traumatic brain damage, Alzheimer’s disease, Parkinson’s disease, multiple sclerosis, psychiatric disorders, autism spectrum disorders, and chronic neuropathic pain. Oxidative stress is a salient and common feature of these conditions and has been strongly implicated in microglial cell activation and neuroinflammation. The transient receptor potential melastatin-related 2 (TRPM2) channel, an oxidative stress-sensitive calcium-permeable cationic channel, is highly expressed in microglial cells. In this review, we examine the recent studies that provide evidence to support an important role for the TRPM2 channel, particularly TRPM2-mediated Ca^2+^ signaling, in mediating microglial cell activation, generation of proinflammatory mediators and neuroinflammation, which are of relevance to CNS pathologies. These findings lead to a growing interest in the TRPM2 channel, a new player in neuroinflammation, as a novel therapeutic target for CNS diseases.

## Introduction

The central nervous system (CNS), which is composed of the brain and spinal cord, is a highly integrated and complex network made up principally by neuronal and glial cells. Neuronal cells or neurons as the working unit of the CNS are specialized to transmit information. Glial cells function more in a supportive capacity to surrounding neurons and, nonetheless, as has been increasingly recognized, also actively participate in many functional aspects of the CNS through bi-directional and dynamic interactions (Jäkel and Dimou, 2017; Allen and Lyons, 2018; Luca et al., 2018). There are several types of glial cells with different embryonic origins (Menassa and Gomez-Nicola, 2018). Astrocytes, oligodendrocytes, and neural-glial antigen 2-positive cells are derived from neuro-ectoderm that also gives rise to neurons, whereas microglial cells are myeloid-lineage cells originated from mesoderm that generates cells of the blood and immune system. Therefore, microglial cells are privileged to be the immune-competent cells of the CNS, like macrophages in the systemic immune system, and thus are often referred to as CNS-resident macrophages. Under healthy or steady-state conditions, microglial cells exhibit a distinctive morphology characteristic of high ramification with an extensive network of fine processes stemming from a small cell body and a resting phenotype (Saijo and Glass, 2011). Microglial cells can secret neurotrophic factors [e.g., brain-derived neurotrophic factor (BDNF)] and, using their phagocytic capability, eliminate excessive or dysfunctional synapses and clear apoptotic developing neurons. In this way, microglial cells support neuronal functions, particularly important processes such as neurogenesis and synaptogenesis during brain development and in the adult brain (Marin-Teva et al., 2004; Sierra et al., 2010; Kettenmann et al., 2013; Yirmiya et al., 2015; Kierdorf and Prinz, 2017; Ising and Heneka, 2018; Luca et al., 2018). In addition, microglial cells act as the sentinel of the CNS and unceasingly patrol the surroundings with their fine processes to monitor environmental changes and provide the first defensive mechanism in response to damage and infection. Microglial cells express a repertoire of the so-called pattern recognition receptors (PRRs), with Toll-like receptors (TLRs), and nucleotide-binding oligomerization domain (NOD)-like receptors (NLRs) being two example groups. PRRs detect danger-associated molecular patterns (DAMPs) released from host cells due to damage or stress or pathogen-associated molecular patterns (PAMPs) generated by invading pathogens (Brubaker et al., 2015; Jassam et al., 2017). Upon ligation of PRRs by DAMPs and/or PAMPs, microglial cells become activated and, after retracting their processes and taking on a spherical form, adopt an amoeboid morphology, proliferate and migrate to the site of damage or infection, where they remove damaged cells or pathogens via phagocytosis (Hanisch and Kettenmann, 2007). Microglial cells can generate proinflammatory mediators that are instrumental in heightening acute immune responses, including chemokines [e.g., C-X-C motif ligand 2 (CXCL2)], cytokines [e.g., interleukin (IL)-1β, tumor necrosis factor (TNF)-α, IL-6], nitric oxide (NO), and reactive oxygen species (ROS). Activated microglial cells can also assume distinctive and anti-inflammatory phenotypes and produce anti-inflammatory cytokines and neurotrophic factors [e.g., IL-10, tissue growth factor (TGF)-β and BDNF] that are important in resolving inflammation and stimulating tissue repair (Wang et al., 2015; Tay et al., 2017; Luca et al., 2018). It is increasingly clear that microglial cells exhibit a high level of heterogeneity in the developing brain and an increase in varied proinflammatory subtypes in the aged, inflamed or neurodegenerative brain (Hammond et al., 2018; Sousa et al., 2018).

It is known that numerous DAMPs are released by cells in the CNS as a result of aging, traumatic damage, chronic psychological stress or neurodegenerative diseases, with ATP being one such well-documented example (Jassam et al., 2017; Wei et al., 2018). It is also well-known that DAMPs are released from degenerating neurons in the brain, such as misfolded amyloid β-peptides (Aβ), α-synuclein, and superoxide dismutase 1 (Glass et al., 2010). These DAMPs are potent inducers of chronical activation or senescence of microglial cells, leading to elevated generation of pro-inflammatory mediators that alters neuronal functions and induces neurotoxicity, a process often referred to as neuroinflammation (Glass et al., 2010; Heneka et al., 2018; Luca et al., 2018). Studies over the past decade have gathered a large body of evidence to support that microglial cells play a key role in mediating neuroinflammation as a significant contributing factor in the progression of aging and a wide spectrum of CNS conditions, including ischemic stroke, traumatic brain damage, Alzheimer’s disease (AD), Parkinson’s disease (AD), multiple sclerosis (MS), amyotrophic lateral sclerosis (ALS), neuropsychiatric disorders [e.g., depression, bipolar disorder (BD), and schizophrenia], autism spectrum disorders (ASD), and neuropathic pain (Glass et al., 2010; Yirmiya et al., 2015; Du et al., 2017; Inoue, 2017; Jassam et al., 2017; Maiti et al., 2017; Ramirez et al., 2017; Salter and Stevens, 2017; Alibhai et al., 2018; Bodnar et al., 2018; Ising and Heneka, 2018; Luca et al., 2018; Shetty et al., 2018; Szepesi et al., 2018; Voet et al., 2018).

Oxidative stress, resulting from excessive ROS generation, impaired antioxidant capacity, or both, is a common and salient feature in aging and the aforementioned CNS diseases. The transient receptor potential melastatin-related 2 (TRPM2) channel is a Ca^2+^-permeable cationic channel with a high sensitivity to oxidative stress or ROS (Hara et al., 2002; Zhang et al., 2003) and is a member of the large transient receptor potential (TRP) superfamily (Clapham, 2003). In the systemic immune system, the TRPM2 channel has been recognized as an important molecular mechanism mediating DAMP/PAMP-induced generation of proinflammatory mediators and innate immune responses (Knowles et al., 2013; Syed Mortadza et al., 2015). Expression of the TRPM2 channel is widely distributed in the CNS with a high level in microglial cells. In this article, we focus on the TRPM2 channel in microglial cells and its role in neuroinflammation. We start with a brief introduction of the TRPM2 channel activation followed by a summary of the evidence supporting TRPM2 channel expression in microglial cells. We proceed to describe the studies that show an important role of the TRPM2 channel in microglial cell activation and generation of proinflammatory mediators in response to various DAMPs and PAMPs, and also the current understanding regarding the molecular mechanisms responsible for DAMP/PAMP-induced TRPM2 channel activation and the downstream TRPM2-dependent signaling pathways engaged in microglial cell activation and generation of proinflammatory mediators. We also discuss the studies using rodent models that demonstrate the role of the TRPM2 channel in microglial cell activation and neuroinflammation in CNS diseases. Finally, we highlight the gaps in our understanding that require further investigation in order to test whether targeting the TRPM2 channel, a new player in neuroinflammation, could represent a neuroprotective approach to tempering the progression of aging or CNS diseases.

## TRPM2 Channel Activation

**FIGURE 1:**
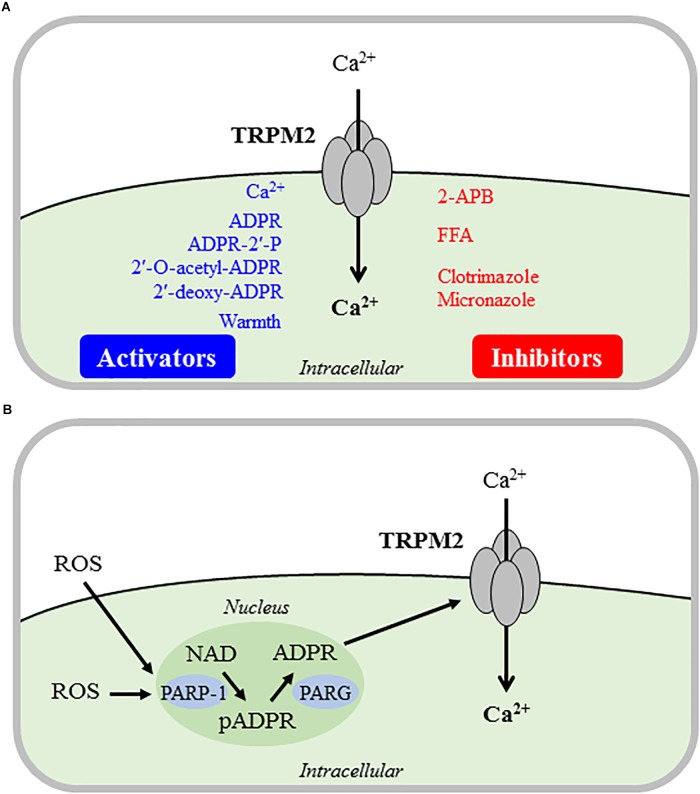
Direct and indirect mechanisms inducing TRPM2 channel activation. Summary of the major mechanisms that are responsible for direct **(A)** or indirect **(B)** activation of the Ca^2+^-permeable TRPM2 channel on the cell surface that mediates Ca^2+^ influx leading to an increase in intracellular Ca^2+^ concentrations. **(A)** Intracellular Ca^2+^, ADPR and several ADPR analogs binds to and activate the TRPM2 channel alone or in synergy. Warm temperature can also induce TRPM2 channel activation in a yet not well-defined mechanism. The TRPM2 channel inhibitors used in the studies discussed in this review are shown here, and note that none of these inhibitors are TRPM2-specific. **(B)** ROS can potently but indirectly induce TRPM2 channel activation, mainly via PARP-1/PARG-mediated ADPR generation from NAD in the nucleus. ADPR, ADP-ribose; ADPR-2′-P, ADPR-2′-phosphate; 2-APB, 2-aminoethyl diphenylborinate; FFA, flufenamic acid; ROS, reactive oxygen species; NAD, nicotinamide adenine dinucleotide; pADPR, poly(ADPR); PARP1, poly(ADPR)-polymerase 1; PARG, poly(ADPR)-glycohydrolase.

Up to now, it has been established both functionally and structurally that the TRPM2 channel is a ligand-gated Ca^2+^-permeable cationic channel activated by intracellular ADP-ribose (ADPR), and that ADPR-induced TRPM2 channel activation displays strong dependence of intracellular Ca^2+^ (Figure 1A) (Perraud et al., 2001; McHugh et al., 2003; Mei et al., 2006; Tong et al., 2006; Xia et al., 2008; Du et al., 2009; Tóth and Csanády, 2010; Huang et al., 2018; Wang et al., 2018; Zhang et al., 2018). Several ADPR analogs, including ADPR-2′-phosphate, 2′-*O*-acetyl-ADPR and 2′-deoxy-ADPR, have been shown to gate the TRPM2 channel (Figure 1A) (Grubisha et al., 2006; Toth et al., 2015; Fliegert et al., 2017). Cyclic ADPR (cADPR), nicotinamide adenine dinucleotide (NAD) and other structurally or metabolically ADPR-related compounds were also reported in earlier studies using whole-cell recording to activate the TRPM2 channel (Sano et al., 2001; Kolisek et al., 2005; Beck et al., 2006; Togashi et al., 2006). This notion however has been challenged by more recent studies using the excised inside-out recording to show that application of these compounds to the intracellular face of the TRPM2 channel failed to induce TRPM2 channel activation (Tóth and Csanády, 2010; Toth et al., 2015).

It is also known that warm temperature (≥35°C) induces TRPM2 channel activation alone or in synergy with other TRPM2 channel activators (Figure 1A), as shown in pancreatic β-cells and macrophages (Togashi et al., 2006; Kashio et al., 2012; Kashio and Tominaga, 2015). In this aspect, recent studies have revealed an important role for the TRPM2 channel in sensory neurons in the peripheral and central nervous systems in detecting non-noxious warmth and regulating body temperature (Song et al., 2016; Tan and McNaughton, 2016).

As introduced above, TRPM2 channels display high sensitivity to activation under oxidative stress or more specifically exposure to elevated levels of ROS, thus gaining increasing recognition for their role in mediating cellular responses to oxidative stress (Jiang et al., 2010; Miller and Zhang, 2011; Takahashi et al., 2011; Knowles et al., 2013; Ru and Yao, 2014; Li et al., 2015, 2017; Syed Mortadza et al., 2015; Yamamoto and Shimizu, 2016). While some earlier studies suggested that ROS such as H_2_O_2_ may directly activate the TRPM2 channel, it is now widely accepted that ROS-induced TRPM2 channel activation is indirect and depends on mechanisms that promote an increase in intracellular ADPR level (Jiang et al., 2010). One widely-employed mechanism in many types of mammalian cells is generation of ADPR from NAD by poly(ADPR)-polymerase (PARP), particularly PARP-1, and poly(ADPR)-glycohydrolase (PARG) in the nucleus (Figure 1B). Some evidence exists to suggest that ADPR generation from NAD catalyzed by NADase in the mitochondria also contributes in ROS-induced TRPM2 channel activation (Perraud et al., 2005).

## TRPM2 Channel Expression in Microglial Cells

Studies examined TRPM2 channel expression in microglial cells at the mRNA, protein and/or functional levels using reverse transcription-polymerase chain reaction (RT-PCR), immunostaining, western blotting, Ca^2+^ imaging and/or patch-clamp current recording (Kraft et al., 2004; Fonfria et al., 2006; Lee et al., 2010; Jeong et al., 2017; Syed Mortadza et al., 2017). Kraft et al. (2004) were the first to examine TRPM2 channel expression in cultured rat microglial cells. A high level of TRPM2 mRNA expression was detected, and exposure to H_2_O_2_ induced extracellular Ca^2+^ influx, leading to an increase in intracellular Ca^2+^ concentration ([Ca^2+^]_i_). Furthermore, application of intracellular ADPR opened a cationic conductance with a linear current-voltage (I-V) relationship and a single channel conductance of ∼65 pS (Kraft et al., 2004), the key biophysical characteristics of the TRPM2 channels (Jiang et al., 2010). A recent study shows strong TRPM2 mRNA and protein expression and ADPR-induced cationic currents in cultured mouse microglial cells (Jeong et al., 2017). Consistently, exposure to H_2_O_2_ (10–300 μM) induced concentration-dependent Ca^2+^ influx and increase in [Ca^2+^]_i_ in cultured mouse microglial cells from wild-type (WT) but not TRPM2-knockout (TRPM2-KO) mice (Syed Mortadza et al., 2017). Profiling the TRPM2 mRNA level in numerous human tissues, including the brain and spinal cord, revealed abundant expression and a wide distribution of TRPM2 expression in the CNS (Fonfria et al., 2006). In C13, a human microglial cell line, TRPM2 mRNA transcripts were also readily detected, and exposure to H_2_O_2_ induced a robust increase in [Ca^2+^]_i_. Both the mRNA expression level and H_2_O_2_-induced Ca^2+^ responses were reduced in C13 cells after treatment with TRPM2-specific antisense oligomers (Fonfria et al., 2006). Furthermore, application of intracellular ADPR or extracellular H_2_O_2_ elicited cationic currents that exhibited an almost linear I-V relationship and a strong sensitivity to inhibition by flufenamic acid (FFA), a TRPM2 channel inhibitor (Figure 1A). In cultured human microglial cells isolated from surgically resected temporal lobe tissues, exposure to H_2_O_2_ elicited a strong increase in [Ca^2+^]_i_ that was inhibited by treatment with clotrimazole (Lee et al., 2010), a TRPM2 channel inhibitor (Figure 1A). These studies have gathered compelling evidence to support TRPM2 channel expression in human and rodent microglial cells as a Ca^2+^ influx pathway with a significant role in ROS-induced Ca^2+^ signaling.

Interestingly, an earlier study noted that there were significantly greater H_2_O_2_-induced Ca^2+^ responses and more readily detectable H_2_O_2_-induced currents in cultured rat microglial cells after exposure to H_2_O_2_ or treatment with lipopolysaccharide (LPS), an endotoxin found in the outer membrane of Gram-negative bacteria and a widely-used PAMP to induce immune cell activation via TLR4 activation (Kraft et al., 2004). TRPM2 mRNA expression was up-regulated in C13 cells after treatment with IL-1β (Fonfria et al., 2006). As we discuss below, exposure to diverse pathological stimuli or conditions can increase TRPM2 channel expression in microglial cells.

## TRPM2 Channel in Microglial Cell Activation and Generation of Proinflammatory Mediators

**FIGURE 2:**
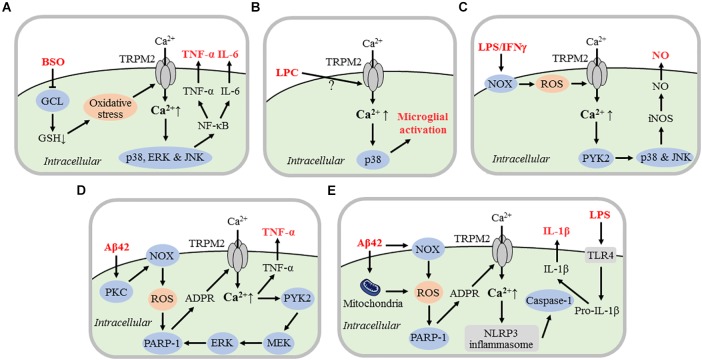
TRPM2 channel mechanisms mediating microglial cell activation and generation of proinflammatory mediators. Schematic illustration of the current knowledge of the signaling mechanisms by which various danger- or pathogen-associated molecular patterns activate the TRPM2 channel in microglial cells, leading to microglial cell activation and generation of proinflammatory mediators. **(A)** BSO-induced GSH depletion via inhibition of GCL-mediated GSH synthesis results in oxidative stress that activates the TRPM2 channel. TRPM2-mediated Ca^2+^ influx induces activation of p38, ERK, and JNK MAPKs and NF-κB pathways that drive expression of TNF-α and IL-6. **(B)** Exposure to LPC induces TRPM2 channel activation via currently unknown mechanisms and ensuring TRPM2-mediated Ca^2+^ influx activates p38, leading to microglial cell activation. **(C)** Exposure to LPS/IFN-γ induces NOX-mediated ROS generation and TRPM2 channel activation. TRPM2-mediated Ca^2+^ influx activates Ca^2+^-sensitive proline rich tyrosine kinase PYK2 and downstream p38 and JNK, triggering iNOS expression and NO generation. **(D)** Exposure to Aβ42 induces TRPM2 channel activation via PKC/NOX-mediated ROS generation, activation of nuclear PARP-1 and ADPR generation. TRPM2-mediated Ca^2+^ and subsequent activation of PYK2 and MEK/ERK serves as a positive feedback mechanism for further TRPM2 channel activation. TRPM2-mediated Ca^2+^ signaling induces TNF-α expression. **(E)** LPS priming of microglial cells promotes expression of biologically inactive pro-IL-1β via TLR4. Exposure to Aβ42 induces mitochondrial and NOX-mediated ROS generation, activation of nuclear PARP-1, and generation of ADPR which opens the TRPM2 channel. TRPM2-mediated Ca^2+^ influx activates NLRP3 inflammasome and subsequently caspase-1. Caspase-1 converts by cleavage pro-IL-1β into biologically active IL-1β. For the evidence that supports or suggests these TRPM2 channel mechanisms in mediating microglial cell activation and generation of proinflammatory mediators, refer to the studies discussed in detail in the text. BSO, D,L-buthionine-*S*,*R*-sulfoximine; GSH, glutathione; GCL, glutamatecysteine ligase; ERK, extracellular signal-regulated kinase; JNK, Jun-N-terminal kinase; MAPK, mitogen-activated protein kinase; TNF-α, tumor-necrosis factor-α; IL, interleukin; LPC, lysophosphatidylcholine; Aβ42, amyloid-β peptide 42; LPS, lipopolysaccharide; IFNγ, interferon γ; NOX; NADPH oxidases; NO, nitric oxide; iNOS, inducible NO synthase; PKC, protein kinase C; PARP-1, poly(ADPR) polymerase 1; TLR4; Toll-like receptor 4; NLRP3, nucleotide binding domain-containing leucine-rich repeat protein 3.

An increasing number of studies have, mainly using cultured microglial cells, investigated the role of the TRPM2 channel in microglial cell activation and generation of proinflammatory mediators in response to diverse pathological stimuli. Furthermore, as discussed in detail next, efforts have been made to gain considerable insights into the mechanisms by which the TRPM2 channel is activated by such stimuli and the downstream TRPM2-dependent signaling pathways in microglial cell activation and generation of proinflammatory mediators (Figure 2).

### Generation of TNF-α and IL-6 Resulting From Impaired Antioxidant Capacity

Glutathione (GSH) is present as one of the important reducing agents in most mammalian tissues including the CNS that equip cells with a non-enzymatic antioxidant capacity (Meister and Anderson, 1983). Glutamatecysteine ligase (or γ-glutamylcysteine synthase) is a rate-limiting step in GSH synthesis and thus D,L-buthionine-*S*,*R*-sulfoximine (BSO), an inhibitor of glutamatecysteine ligase, can cause depletion of intracellular GSH and cellular oxidative stress. It has been proposed that a reduction in intracellular GSH with aging increases age-related susceptibility to oxidative stress, which is worsened in many neurodegenerative conditions (Sohal and Weindruch, 1996). A previous study investigated the role of the TRPM2 channel in generating neurotoxic proinflammatory mediators in cultured human microglial cells under BSO-induced oxidative stress (Lee et al., 2010). Exposure to BSO (1–24 h) induced an exposure duration-dependent increase in [Ca^2+^]_i_. Exposure to BSO for 2 h was sufficient to activate mitogen-activated protein kinases (MAPK), p38, extracellular signal-regulated kinase (ERK) and Jun-N-terminal kinase (JNK), and furthermore downstream nuclear factor NF-κB. BSO-induced increase in [Ca^2+^]_i_ and activation of MAPK and NF-κB signaling pathways were significantly suppressed by supplementation with GSH or treatment with clotrimazole. Exposure to BSO (0.1, 0.5 and 1 mM) also induced concentration-dependent release of TNF-α and IL-6 from microglial cells, which was reduced by treatment with TRPM2-specific small interference RNA (siRNA) (Lee et al., 2010). These results suggest that oxidative stress resulting from GSH depletion activates the TRPM2 channel and TRPM2-mediated Ca^2+^ influx in turn initiates downstream MAPK and NF-kB signaling pathways, leading to generation of TNF-α and IL-6 (Figure 2A). Human neuroblastoma SH-SY5Y cells cultured in the medium conditioned by BSO-treated microglial cells exhibited substantial cell death (Lee et al., 2010). Such cell death was significantly attenuated in the conditioned culture medium that was prior depleted of TNF-α and IL-6. Moreover, SH-SY5Y cell death in the conditioned culture medium was strongly suppressed by supplementing microglial cell culture medium with GSH or treating microglial cells with clotrimazole or TRPM2-siRNA (Lee et al., 2010). Collectively, these results suggest that TNF-α and IL-6, generated by microglial cells in a TRPM2-dependent manner, under BSO-induced oxidative stress can induce neurotoxicity.

### LPC-Induced Microglial Cell Activation

It is known that lysophosphatidylcholine (LPC), an inflammatory phospholipid endogenously generated under physiological and various pathological conditions, can induce extracellular Ca^2+^ influx in microglial cells and microglial cell activation (Schilling et al., 2004; Sheikh et al., 2009). A recent study has investigated the role of the TRPM2 channel in LPC-induced Ca^2+^-signaling and microglial cell activation in cultured mouse microglial cells (Jeong et al., 2017). Exposure to LPC induced cationic currents as well as an extracellular Ca^2+^-dependent increase in [Ca^2+^]_i_. LPC exposure also resulted in phosphorylation of p38 (p-p38), an indicator of microglial cell activation. Consistently, intrathecal injection of LPC enhanced expression of ionized calcium binding adapter molecule 1 (Iba1) and CD11 in spinal microglial cells, suggesting microglial cell activation (Jeong et al., 2017). Such LPC-induced *in vitro* or *in vivo* effects in microglial cells were largely prevented by TRPM2-KO (Jeong et al., 2017). These results support a key role for the TRPM2 channel in LPC-induced Ca^2+^ signaling and activation of downstream p38 MAPK signaling pathways, leading to microglial cell activation (Jeong et al., 2017) (Figure 2B). It remains unclear regarding the mechanisms by which LPC induces TRPM2 channel activation, and the types of proinflammatory mediators that are generated as a result of LPC-induced microglial cell activation. This study has made an interesting observation that the levels of both total and cell surface TRPM2 protein expression was significantly increased in LPC-treated microglial cells but it is not elucidated how such up-regulation of TRPM2 expression and membrane trafficking occurs.

### LPS/IFNγ-Induced Activation of iNOS and Generation of NO

The TRPM2 channel was shown, in an *in vivo* study discussed below, to play a significant role in mediating spinal microglial cell activation and neuropathic pain (Haraguchi et al., 2012). In this study the authors particularly revealed a role for the TRPM2 channel in cultured microglial cells in the activation of inducible NO synthase (iNOS) and generation of NO after exposure to LPS and IFNγ. A subsequent study by the same group investigated the signaling pathways engaged in LPS/IFNγ-induced TRPM2 channel activation and NO generation (Miyake et al., 2014). LPS/IFNγ exposure evoked extracellular Ca^2+^ influx to increase [Ca^2+^]_i_, which was prevented by TRPM2-KO or treatment with miconazole, a TRPM2 channel inhibitor (Figure 1A). Such Ca^2+^ response was also efficiently inhibited by treatment with diphenylene iodonium (DPI) and ML-171, inhibitors of nicotinamide adenine dinucleotide phosphate (NADPH)-dependent oxidases (NOXs). LPS/IFNγ-induced NO generation was also significantly reduced by TRPM2-KO, or by inclusion of 1,2*-bis*(*o*-aminophenoxy)ethane-*N,N,N,N*-tetraacetic acid (BAPTA), a Ca^2+^ chelator, to remove extracellular Ca^2+^. These results support that LPS/IFNγ induce NOX-mediated ROS generation, TRPM2 channel activation and an increase in [Ca^2+^]_i,_ leading to NO generation (Figure 2C). Moreover, LPS/IFNγ-induced NO generation was attenuated by treatment with AG17, an inhibitor for Ca^2+^-sensitive proline-rich tyrosine kinase 2 (PYK2), SB203580, a p38 inhibitor, or SP600125, a JNK inhibitor. Inhibition of LPS/IFNγ-induced NO generation by BAPTA, AG17, SB203580 or SP600125 was abolished by TRPM2-KO. LPS/IFNγ-induced NO generation in microglial cells from both WT and TRPM2-KO mice was attenuated by treatment with PD98059, a MEK/ERK inhibitor. Likewise, exposure to LPS/IFNγ induced selective activation of p38 in WT but not TRPM2-KO microglial cells, but indiscriminate activation of ERK in both WT and TRPM2-KO microglial cells. Overall, these results suggest that LPS/IFNγ-induced TRPM2-mediated Ca^2+^ signaling initiates activation of PYK2 and downstream p38/JNK MAPK signaling pathways for activation of iNOS and subsequent NO generation (Figure 2C).

### Aβ42-Induced Microglial Cell Activation and Generation of TNF-α

A recent *in vivo* study using the APP/PS1 mouse model of AD, as discussed further below, has disclosed an important role of the TRPM2 channel in Aβ-induced AD pathologies, including microglial cell activation (Ostapchenko et al., 2015). It is well-established that TNF-α contributes to AD and neurodegenerative diseases via direct interaction with its death receptor on neurons as well as induction of microglial cell activation to generate additional neurotoxic mediators (Alam et al., 2016; Jiang et al., 2018). Our recent study has explored the molecular mechanisms responsible for TRPM2 channel activation and TNF-α generation in cultured mouse microglial cells induced by exposure to Aβ42, one of the amyloid-β peptides of high relevance to AD (Syed Mortadza et al., 2018). Exposure to Aβ42 (30–300 nM) induced a concentration-dependent and extracellular Ca^2+^-dependent increase in [Ca^2+^]_i_. Aβ42-induced Ca^2+^ response was strongly suppressed by treatment with 2-APB, a TRPM2 channel inhibitor (Figure 1), or BAPTA-AM as a membrane-permeable and thus intracellular Ca^2+^ chelator, and furthermore by TRPM2-KO. Exposure to Aβ42 induced cellular ROS generation and activation of nuclear PARP-1. Both Aβ42-induced PARP-1 activation and increase in [Ca^2+^]_i_ were suppressed by treatment with PJ34, an inhibitor of PARP enzymes including PARP-1. Furthermore, Aβ42-induced ROS generation, PARP-1 activation and Ca^2+^ responses were inhibited by treatment with chelerythrine, a protein kinase C (PKC) inhibitor, GKT137831, a NOX1/4-seletive inhibitor, or Phox-I2, a NOX2 inhibitor as well as the NOX generic inhibitor DPI. These results indicate that Aβ42 activates the TRPM2 channel by inducing PKC/NOX-mediated ROS generation and subsequent PARP-1 activation and generation of ADPR (Figure 2D). Aβ42-induced PARP-1 activation and increase in [Ca^2+^]_i_ were also prevented by treatment with PF431396, a PYK2 inhibitor, or U0126, a MEK/ERK inhibitor. Aβ42-induced PARP-1 activation was significantly reduced but incompletely abolished by TRPM2-KO, and the remaining Aβ42-induced PARP-1 activity in TRPM2-KO microglial cells was prevented by treatment with GKT137831 or Phox-I2 and, in striking contrast, not altered by treatment with PF431396 or U0126. Taken together, these results suggest that Aβ42 stimulates PKC/NOX-mediated ROS generation and PARP-1 activation leading to initial TRPM2 channel activation, and that subsequent TRPM2-mediated Ca^2+^ flux and activation of PYK2, MEK/ERK, and PARP-1 serves as a positive feedback mechanism for further TRPM2 channel activation (Figure 2D). Moreover, exposure to Aβ42 induced noticeable morphological changes in microglial cells and an increase in the expression and release of TNF-α. Aβ42-induced morphological changes and TNF-α generation were prevented by TRPM2-KO and, moreover, by pharmacological inhibition of the aforementioned signaling pathways responsible for TRPM2 channel activation (Syed Mortadza et al., 2018).

### Aβ42-Induced Activation of NLRP3 Inflammasome and Generation of IL-1β

The nucleotide binding domain-containing leucine-rich repeat protein 3 (NLRP3) is a member of the NOD family of PRRs in the cytosol. In response to damage or infection, NLRP3, apoptosis-associated speck-like protein containing a caspase recruitment domain, and procaspase-1, via protein-protein interactions, assemble a multi-protein complex often termed as the NLRP3 inflammasome. NLRP3 inflammasome activation is required to activate caspase-1, which in turn cleaves pro-IL-1β into IL-1β (Tschopp and Schroder, 2010; Brubaker et al., 2015; Jassam et al., 2017; Song et al., 2017; White et al., 2017). It was shown that genetic inactivation of the NLRP3 inflammasome in APP/PS1 mice reduced IL-1β generation by microglial cells, leading to improved spatial memory and attenuation of other AD-related pathological phenotypes (Heneka et al., 2013). In addition, NLRP3 inflammasome inactivation shifted microglial cells toward an anti-inflammatory phenotype that cleared Aβ peptides, thereby resulting in a reduction in amyloid-β deposition (Heneka et al., 2013). Therefore, neuroinflammation resulting from NLPR3 inflammasome activation and IL-1β generation in microglial cells has emerged as an important factor contributing to AD pathogenesis, inciting an interest in targeting the NLRP3 inflammasome as a therapeutic approach to AD (Heneka et al., 2014; White et al., 2017). It is well-known that NLRP3 inflammasome activation and IL-1β generation in immune cells including microglial cells exhibit a striking convergence on ROS generation (Tschopp and Schroder, 2010; Song et al., 2017). A recent pharmacological study has examined the potential role of the TRPM2 channel in Aβ42-induced NLRP3 inflammasome activation and IL-1β generation in LPS-primed microglial cells (Aminzadeh et al., 2018). Exposure to Aβ42 at a relatively high concentration (10 μM) induced mitochondrial ROS generation and also IL-1β generation, both of which were suppressed by treatment with DPI at a high concentration (20 μM) that presumably targets mitochondrial ROS generation. Aβ42-induced IL-1β generation was inhibited by treatment with VAS2870 or (2R,4R)-4-aminopyrrolidine-2,4-dicarboxylate, NOX inhibitors, indicating engagement of NOX-meditated ROS generation. In addition, Aβ42-induced IL-1β generation was reduced by treatment with *N*-acetylcysteine, a ROS scavenger, or DPQ, a PARP-1 inhibitor (Aminzadeh et al., 2018). Exposure to Aβ42 resulted in a Ca^2+^ influx-dependent increase in [Ca^2+^]_i_ that was also strongly inhibited by treatment with DPI, VAS2870, DPQ, or BAPTA-AM. Finally, Aβ42-induced caspase-1 activation, as shown by western blotting, was inhibited by treatment with DPQ or BAPTA-AM (Aminzadeh et al., 2018). These results are consistent with the notion that Aβ42 induces NLRP3 inflammasome activation and IL-1β generation via stimulating mitochondrial and NOX-mediated ROS generation, activation of PARP-1 and the TRPM2 channel, and subsequent TRPM2-mediated Ca^2+^ influx (Figure 2E). However, more and definitive evidence is required to corroborate the proposed role of the TRPM2 channel.

## TRPM2 Channel in Neuroinflammation and CNS Pathologies

**FIGURE 3:**
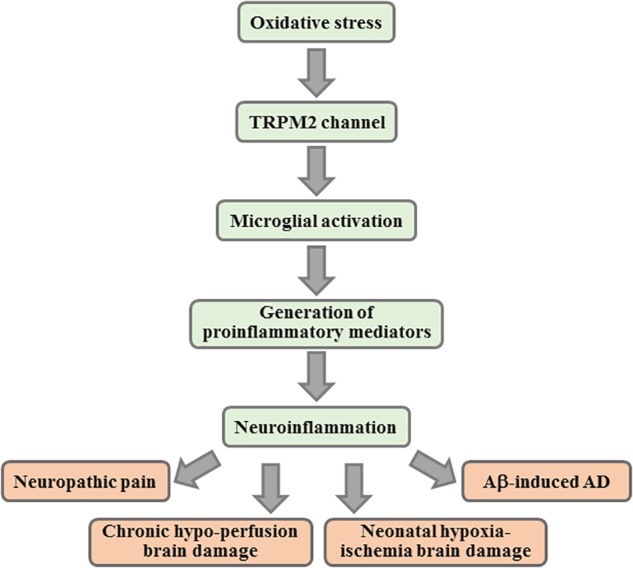
Contribution of TRPM2 channel-mediated neuroinflammation in CNS pathologies. Summary of the key events in TRPM2-mediated neuroinflammation implicated in various CNS pathologies. Activation of the TRPM2 channel in microglial cells mediates microglial cell activation, generation of proinflammatory mediators and/or neuroinflammation that have been shown to contribute to the pathogenesis of neuropathic pain, brain damage by chronic cerebral hypo-perfusion and neonatal hypoxia-ischemia, and Aβ-induced AD. CNS, central nervous system; Aβ, amyloid β peptides; AD, Alzheimer’s disease.

It is clear from the above discussion that studies based on cultured microglial cells support an important role for the TRPM2 channel in microglial cell activation and generation of neurotoxic proinflammatory mediators in response to DAMPs/PAMPs of high relevance to various CNS diseases. As discussed next, there is increasing evidence from *in vivo* studies using rodent models that supports a critical role for the TRPM2 channel in microglial cells in microglial cell activation, generation of proinflammatory mediators and neuroinflammation in the pathogenesis of CNS diseases (Figure 3).

### Neuropathic Pain

It is well-recognized that microglial cell activation in the spinal cord, as well as peripheral neuroinflammation, plays a significant role in the development of chronic neuropathic pain (Ji and Suter, 2007; Costigan et al., 2009; Tsuda et al., 2013). The role of the TRPM2 channel in mediating spinal microglial cell activation and neuropathic pain was explored in a previous study using two mouse models of neuropathic pain induced by partial sciatic nerve ligation (SNL) and spinal nerve transection (SNT), respectively (Haraguchi et al., 2012). Both mechanical allodynia and thermal hyperalgesia observed in WT mice during the 2 weeks following SNL were largely absent in TRPM2-KO mice. In the sciatic nerves on the ligation site, the TRPM2 mRNA level was markedly elevated. SNL induced a significant increase in the number of neutrophils and also in generation of CXCL2 in WT mice, both of which were mitigated or completely prevented in TRPM2-KO mice. SNL also resulted in a strong increase in the TRPM2 mRNA expression in dorsal microglial cells and in the intensity of immunoreactivity for both Iba1 and CD11b. Moreover, there was an increase in p-p38 and strong co-localization of p-p38 and CD11b, further indicating microglial cell activation. SNL-induced increase in the intensity of immunoreactivity for Iba1, CD11b nd p-p38, and co-localization of p-p38 and CD11b in spinal microglial cells was largely prevented by TRPM2-KO. Similarly, SNT induced mechanical allodynia, increased intensity of immunoreactivity to CD11b and p-p38 and their co-localization in dorsal microglial cells, all of which were significantly subdued in TRPM2-KO mice. The study further examined the role of the TRPM2 channel in the generation of proinflammatory mediators in cultured microglial cells exposed to LPS/IFNγ. LPS/IFNγ stimulated CXCL2 generation and NO release as well as an increase in the mRNA expression of CXCL2, TNF-α, IL-1β, IL-6, and iNOS. LPS/IFNγ-induced generation of CXCL2 and NO, and increased mRNA expression of CXCL2 and iNOS were significantly lowered by TRPM2-KO (Haraguchi et al., 2012). However, the study revealed no significant effect of TRPM2-KO on the mRNA expression of TNF-α, IL-1β and IL-6, indicating engagement of TRPM2-independent mechanisms. These results support the notion that the TRPM2 channel in spinal microglial cells contributes to neuropathic pain by mediating the generation of proinflammatory mediators to aggravate pro-nociceptive inflammatory responses. As discussed above, LPS/IFNγ-induced NO generation depends on TRPM2-mediated Ca^2+^ signaling and activation of downstream PYK2 and MAPK p38 and JNK signaling pathways (Figure 2C).

### Alzheimer’s Disease

Alzheimer’s disease is an age-related neurodegenerative disease with increasing prevalence in a rapidly aging society, representing the most common cause of dementia that afflicts tens of millions of older people worldwide. Aβ accumulation is widely thought to be an early and pathogenic event in AD pathogenesis. Oxidative damage is a conspicuous but mechanistically poorly understood feature of AD. As has been recently reviewed (Jiang et al., 2018), studies have shown wide expression of the TRPM2 channel in the brain and strong evidence for the TRPM2 channel as a nexus from Aβ generation and oxidative damage to AD pathologies via multiple cellular and molecular mechanisms, including microglial cell activation. Microglial cells are known to have a dual role in AD (Boche and Nicoll, 2008). They provide a protective role by phagocytic clearance of Aβ, but such a beneficial capacity declines with aging and is overwhelmed by excessive toxic aggregates, becoming inefficient. As introduced above, Aβ can induce chronic activation and senescence of microglial cells leading to excessive generation of ROS and numerous neurotoxic proinflammatory cytokines, such as TNF-α, IL-1β and IL-6, which constitutes a critical component of AD pathogenesis. APP/PS1 mice co-express a chimeric mouse/human amyloid precursor protein (APP) with the Swedish mutations (K670N and M671L) and human presenilin 1 (PS1) with deletion of exon 9 (Jankowsky et al., 2003). A recent study has examined the role of the TRPM2 channel in Aβ-induced AD pathogenesis using this mouse AD model (Ostapchenko et al., 2015). As has been well-documented, the APP/PS1 mice exhibit excessive Aβ generation, amyloid deposits and synaptic loss in the hippocampus and cortex, microglial cell activation, and severe impairment in age-related spatial memory. Genetic deletion of TRPM2 expression in APP/PS1 mice, while resulting in no alteration in amyloid deposition, essentially reversed Aβ-induced synaptic loss, microglial cell activation, and memory impairment (Ostapchenko et al., 2015). These results provide compelling evidence to support a critical role for the TRPM2 channel in Aβ-induced AD-related pathologies. As already discussed above, recent *in vitro* studies reveal an important role of the TRPM2 channel in mediating Aβ42-induced microglial cell activation and generation of TNF-α (Syed Mortadza et al., 2018) and possibly IL-1β (Aminzadeh et al., 2018).

### Brain Damage by Deficient Cerebral Blood Circulation

The brain is well-known for its vulnerability to damage by deprivation or restriction of oxygen and/or glucose supply that can occur under conditions such as cerebral ischemic stroke, cardiac arrest, chronic cerebral hypo-perfusion, and neonatal hypoxia-ischemia. Oxidative stress, mainly due to increased ROS generation, is a common characteristic of these conditions. An early study demonstrated elevated TRPM2 mRNA expression in rat brains at 1 and 4 weeks after transient middle cerebral artery occlusion (MCAO), a widely-used rodent model of ischemic stroke (Fonfria et al., 2006). A number of recent studies, using various *in vitro* and *in vivo* mouse models of ischemia-reperfusion in conjunction with pharmacological inhibition or genetic deletion of the TRPM2 channel, have supported a critical role of the TRPM2 channel in ischemia-reperfusion brain damage and associated cognitive dysfunction (Jia et al., 2011; Alim et al., 2013; Shimizu et al., 2013, 2016; Gelderblom et al., 2014; Ye et al., 2014). There is also emerging evidence to indicate a role for the TRPM2 channel in mediating brain damage due to hypoxia-ischemia in neonates (Huang et al., 2017) and chronic cerebral hypo-perfusion in adults (Miyanohara et al., 2018).

Of notice, much of the research in this area has so far been devoted to the TRPM2 channel in mediating neuronal death. Nonetheless, there is increasing evidence to suggest a significant contribution of TRPM2-mediated neuroinflammation. For example, selective deletion of the TRPM2 expression in peripheral immune cells substantially protected infarction and cognitive impairment in mice after transient MCAO and reperfusion (Gelderblom et al., 2014). However, the role of the TRPM2 channel in microglial cells in ischemia-reperfusion brain damage largely remains unclear. In the case of neonatal hypoxia-ischemia, a recent study shows that infarction in postnatal day 7 pups, induced by ligating the right common carotid artery and reducing oxygen supply and examined 24 h or 7 days afterward, was considerably attenuated in heterozygous and homozygous TRPM2-KO pups. In addition, WT pups exhibited sensorimotor dysfunction at 7 days post hypoxia-ischemia, and such deficits were less noticeable in heterozygous and homozygous TRPM2-KO pups. In WT pups, the TRPM2 mRNA expression was greater in the damaged hemisphere than the healthy hemisphere. Hypoxia-ischemia induced a massive increase in the number of glial fibrillary acidic protein (GFAP) positive cells and Iba1-positive cells in WT pups, but not in heterozygous and homozygous TRPM2-KO pups. These results suggest that the TRPM2 channel plays an important role in mediating activation of glial cells, including microglial cells, thereby inducing neonatal hypoxic-ischemic brain damage. The role of the TRPM2 channel in microglial cells has been best understood in brain damage by chronic cerebral hypo-perfusion (Miyanohara et al., 2018). Mice manifested significant white matter damage and cognitive dysfunction 28 days after introduction of bilateral common carotid artery stenosis (BCAS), a model of chronic cerebral hypo-perfusion. At this time point, there was also a significant increase in the TRPM2 mRNA expression and in the level of IL-1β, TNF-α and IL-6 in the corpus callosum. Such BCAS-induced effects, namely, white matter damage, cognitive dysfunction and increased generation of IL-1β, TNF-α and IL-6, were prevented by TRPM2-KO. There was an increase in the number of GFAP positive cells and Iba-1 positive cells in the corpus callosum at 14 and 28 days after BCAS, but only the number of Iba-1 positive cells was strongly suppressed by TRPM2-KO. The increase in the number of Iba1-positive cells and cognitive dysfunction in BCAS-operated mice was effectively prevented by administration of minocycline, an inhibitor of microglial cell and macrophage activation. Further analysis, using WT and TRPM2-KO mice with bone marrow (BM)-derived cells replaced by WT GFP-labeled BM-derived cells, indicates that the Iba-1 positive cells in white matter mainly are largely microglial cells. Collectively, these results therefore support a critical role for the TRPM2 channel in mediating microglial cell activation and generation of proinflammatory cytokines, IL-1β, TNF-α and IL-6, in the aggravation of cognitive impairment by chronic cerebral hypo-perfusion.

## Summary and Perspectives

In summary, the TRPM2 channel is highly expressed in microglial cells and mainly functions as a plasma membrane Ca^2+^-permeable cationic channel with a key role in mediating ROS-induced Ca^2+^ signaling (Figure 1B). In addition, the TRPM2 channel in microglial cells is potently activated by diverse DAMPs and/or PAMPs that induce mitochondrial and/or NOX-mediated ROS generation, activation of PARP-1 and ADPR generation (Figure 2). Studies using rodent models in combination with pharmacological and genetic interventions support a significant role for the TRPM2 channel in microglial cell activation and neuroinflammation in the pathogenesis of various CNS conditions. Currently, this includes neuropathic pain, chronic cerebral hypo-perfusion brain damage, neonatal hypoxia-ischemia and Aβ-induced AD (Figure 3). As mentioned in the introduction, microglial cell-mediated neuroinflammation is a well-recognized factor in the pathogenesis of many other CNS conditions besides the aforementioned conditions. Research has also implicated TRPM2 channel in PD (Sun et al., 2018; An et al., 2019; Li and Jiang, 2019), MS (Tsutsui et al., 2018), traumatic brain damage (Cook et al., 2010; Yürüker et al., 2015), and neurodevelopmental disorders such as ASD (Higashida et al., 2018) and depression (Xu et al., 2006; Jang et al., 2015; Zhong et al., 2016; Ko et al., 2019) as well as ischemic stroke brain damage. Evidently, further research is required to investigate whether the TRPM2 channel in microglial cells in mediating neuroinflammation plays a significant role in these CNS conditions.

As discussed above, recent studies have gained significant insights into the molecular mechanisms by which DAMPs and/or PAMPs induce activation of the TRPM2 channel and generation of diverse proinflammatory mediators that are of strong relevance to various CNS diseases. It is clear from the discussion that the current understanding remains fragmented with better insights in some cases than others (Figure 2). Further research is required to provide a coherent understanding of how the TRPM2 channel is activated in response to distinctive stimuli or under different conditions, leading to activation of downstream Ca^2+^ signaling pathways, and ultimately how such TRPM2-dependent signaling pathways drive microglial cell activation and generation of proinflammatory mediators.

Given the widespread indication of a significant role for the TRPM2 channel in mediating neuroinflammation and CNS diseases, the TRPM2 channel represents an attractive therapeutic target. The TRPM2 channel also plays important roles in a number of physiological processes, such as insulin release from pancreatic β-cells, regulation of temperature sensation, and peripheral immune responses, which may complicate the concept of targeting TRPM2 as a therapeutic strategy. However, TRPM2 channel expression in the CNS is selectively up-regulated by diverse pathological stimuli or diseased conditions. With continual research into TRPM2 modulation and function in specific cell types, future developments may focus on pharmacological agents that can improve the outcome for patients with CNS diseases while sparing the physiological functions of the channel. Targeting the TRPM2 channel in microglial cells, a newly-emerged player in neuroinflammation, represents an interesting a venue of development of promising therapeutics.

## Author Contributions

L-HJ and PM wrote the manuscript. All the authors contributed to literature research and analysis, developed the review topic, and approved the manuscript.

## Conflict of Interest Statement

The authors declare that the research was conducted in the absence of any commercial or financial relationships that could be construed as a potential conflict of interest.

## References

[B1] Alam Q. Alam M. Z. Mushtaq G. Damanhouri G. A. Rasool M. Kamal M. A. (2016). Inflammatory process in Alzheimer’s and parkinson’s diseases: central role of cytokines. *Curr. Pharm. Des.* 22 541–548. 10.2174/1381612822666151125000300 26601965

[B2] Alibhai J. D. Diack A. B. Manson J. C. (2018). Unravelling the glial response in the pathogenesis of Alzheimer’s disease. *FASEB J.* 32 5766–5777. 10.1096/fj.201801360R 30376380

[B3] Alim I. Teves L. Li R. Mori Y. Tymianski M. (2013). Modulation of NMDAR subunit expression by TRPM2 channels regulates neuronal vulnerability to ischemic cell death. *J. Neurosci.* 33 17264–17277. 10.1523/JNEUROSCI.1729-13.2013 24174660 PMC6618359

[B4] Allen N. J. Lyons D. A. (2018). Glia as architects of central nervous system formation and function. *Science* 362 181–185. 10.1126/science.aat0473 30309945 PMC6292669

[B5] Aminzadeh M. Roghani M. Sarfallah A. Riazi G. H. (2018). TRPM2 dependence of ROS-induced NLRP3 activation in Alzheimer’s disease. *Int. Immunopharmacol.* 54 78–85. 10.1016/j.intimp.2017.10.024 29107864

[B6] An X. Fu Z. Mai C. Wang W. Wei L. Li D. (2019). Increasing the TRPM2 channel expression in human neuroblastoma SH-SY5Y cells augments the susceptibility to ROS-induced cell death. *Cells.* 8:E28. 10.3390/cells8010028 30625984 PMC6356620

[B7] Beck A. Kolisek M. Bagley L. A. Fleig A. Penner R. (2006). Nicotinic acid adenine dinucleotide phosphate and cyclic ADP-ribose regulate TRPM2 channels in T lymphocytes. *FASEB J.* 20 962–964. 10.1096/fj.05-5538fje 16585058

[B8] Boche D. Nicoll J. A. (2008). The role of the immune system in clearance of Abeta from the brain. *Brain Pathol.* 18 267–278. 10.1111/j.1750-3639.2008.00134.x 18363937 PMC8095633

[B9] Bodnar C. N. Morganti J. M. Bachstetter A. D. (2018). Depression following a traumatic brain injury: uncovering cytokine dysregulation as a pathogenic mechanism. *Neural. Regen. Res.* 13 1693–1704. 10.4103/1673-5374.238604 30136679 PMC6128046

[B10] Brubaker S. W. Bonham K. S. Zanoni I. Kagan J. C. (2015). Innate immune pattern recognition: a cell biological perspective. *Annu. Rev. Immunol.* 33 257–290. 10.1146/annurev-immunol-032414-112240 25581309 PMC5146691

[B11] Clapham D. E. (2003). TRP channels as cellular sensors. *Nature* 426 517–524. 10.1038/nature02196 14654832

[B12] Cook N. L. Vink R. Helps S. C. Manavis J. Van Den Heuvel C. (2010). Transient receptor potential melastatin 2 expression is increased following experimental traumatic brain injury in rats. *J. Mol. Neurosci.* 42 192–199. 10.1007/s12031-010-9347-8 20309649

[B13] Costigan M. Scholz J. Woolf C. J. (2009). Neuropathic pain: a maladaptive response of the nervous system to damage. *Annu. Rev. Neurosci.* 32 1–32. 10.1146/annurev.neuro.051508.135531 19400724 PMC2768555

[B14] Du J. Xie J. Yue L. (2009). Intracellular calcium activates TRPM2 and its alternative spliced isoforms. *Proc. Natl. Acad. Sci. U.S.A.* 106 7239–7244. 10.1073/pnas.0811725106 19372375 PMC2678461

[B15] Du L. Zhang Y. Chen Y. Zhu J. Yang Y. Zhang H. L. (2017). Role of microglia in neurological disorders and their potentials as a therapeutic target. *Mol. Neurobiol.* 54 7567–7584. 10.1007/s12035-016-0245-0 27830532

[B16] Fliegert R. Watt J. M. Schobel A. Rozewitz M. D. Moreau C. Kirchberger T. (2017). Ligand-induced activation of human TRPM2 requires the terminal ribose of ADPR and involves Arg1433 and Tyr1349. *Biochem. J.* 474 2159–2175. 10.1042/BCJ20170091 28515263 PMC5473349

[B17] Fonfria E. Murdock P. R. Cusdin F. S. Benham C. D. Kelsell R. E. McNulty S. (2006). Tissue distribution profiles of the human TRPM cation channel family. *J. Recept. Signal Transduct. Res.* 26 159–178. 10.1080/10799890600637506 16777713

[B18] Gelderblom M. Melzer N. Schattling B. Göb E. Hicking G. Arunachalam P. (2014). Transient receptor potential melastatin subfamily member 2 cation channel regulates detrimental immune cell invasion in ischemic stroke. *Stroke* 45 3395–3402. 10.1161/STROKEAHA.114.005836 25236871

[B19] Glass C. K. Saijo K. Winner B. Marchetto M. C. Gage F. H. (2010). Mechanisms underlying inflammation in neurodegeneration. *Cell* 140 918–934. 10.1016/j.cell.2010.02.016 20303880 PMC2873093

[B20] Grubisha O. Rafty L. A. Takanishi C. L. Xu X. Tong L. Perraud A. L. (2006). Metabolite of SIR2 reaction modulates TRPM2 ion channel. *J. Biol. Chem.* 281 14057–14065. 10.1074/jbc.M513741200 16565078

[B21] Hammond T. R. Robinton D. Stevens B. (2018). Microglia and the brain: complementary partners in development and disease. *Annu. Rev. Cell Dev. Biol.* 34 523–544. 10.1146/annurev-cellbio-100616-060509 30089221

[B22] Hanisch U. K. Kettenmann H. (2007). Microglia: active sensor and versatile effector cells in the normal and pathologic brain. *Nat. Neurosci.* 10 1387–1394. 10.1038/nn1997 17965659

[B23] Hara Y. Wakamori M. Ishii M. Maeno E. Nishida M. Yoshida T. (2002). LTRPC2 Ca^2+^-permeable channel activated by changes in redox status confers susceptibility to cell death. *Mol. Cell* 9 163–173. 10.1016/S1097-2765(01)00438-5 11804595

[B24] Haraguchi K. Kawamoto A. Isami K. Maeda S. Kusano A. Asakura K. (2012). TRPM2 contributes to inflammatory and neuropathic pain through the aggravation of pronociceptive inflammatory responses in mice. *J. Neurosci.* 32 3931–3941. 10.1523/JNEUROSCI.4703-11.2012 22423113 PMC6703465

[B25] Heneka M. T. Kummer M. P. Latz E. (2014). Innate immune activation in neurodegenerative disease. *Nat. Rev. Immunol.* 14 463–477. 10.1038/nri3705 24962261

[B26] Heneka M. T. Kummer M. P. Stutz A. Delekate A. Schwartz S. Vieira-Saecker A. (2013). NLRP3 is activated in Alzheimer’s disease and contributes to pathology in APP/PS1 mice. *Nature* 493 674–678. 10.1038/nature11729 23254930 PMC3812809

[B27] Heneka M. T. Mcmanus R. M. Latz E. (2018). Inflammasome signalling in brain function and neurodegenerative disease. *Nat. Rev. Neurosci.* 19 610–621. 10.1038/s41583-018-0055-7 30206330

[B28] Higashida H. Yuhi T. Akther S. Amina S. Zhong J. Liang M. (2018). Oxytocin release via activation of TRPM2 and CD38 in the hypothalamus during hyperthermia in mice: implication for autism spectrum disorder. *Neurochem. Int.* 119 42–48. 10.1016/j.neuint.2017.07.009 28736241

[B29] Huang S. Turlova E. Li F. Bao M. H. Szeto V. Wong R. (2017). Transient receptor potential melastatin 2 channels (TRPM2) mediate neonatal hypoxic-ischemic brain injury in mice. *Exp. Neurol.* 296 32–40. 10.1016/j.expneurol.2017.06.023 28668375

[B30] Huang Y. Winkler P. A. Sun W. Lu W. Du J. (2018). Architecture of the TRPM2 channel and its activation mechanism by ADP-ribose and calcium. *Nature* 562 145–149. 10.1038/s41586-018-0558-4 30250252

[B31] Inoue K. (2017). Purinergic signaling in microglia in the pathogenesis of neuropathic pain. *Proc. Jpn. Acad. Ser. B. Phys. Biol. Sci.* 93 174–182. 10.2183/pjab.93.011 28413195 PMC5489427

[B32] Ising C. Heneka M. T. (2018). Functional and structural damage of neurons by innate immune mechanisms during neurodegeneration. *Cell Death Dis.* 9:120. 10.1038/s41419-017-0153-x 29371603 PMC5833757

[B33] Jäkel S. Dimou L. (2017). Glial cells and their function in the adult brain: a journey through the history of their ablation. *Front. Cell Neurosci.* 11:24. 10.3389/fncel.2017.00024 28243193 PMC5303749

[B34] Jang Y. Lee S. H. Lee B. Jung S. Khalid A. Uchida K. (2015). TRPM2, a susceptibility gene for bipolar disorder, regulates glycogen synthase kinase-3 activity in the brain. *J. Neurosci.* 35 11811–11823. 10.1523/JNEUROSCI.5251-14.2015 26311765 PMC6705460

[B35] Jankowsky J. L. Xu G. Fromholt D. Gonzales V. Borchelt D. R. (2003). Environmental enrichment exacerbates amyloid plaque formation in a transgenic mouse model of Alzheimer disease. *J. Neuropathol. Exp. Neurol.* 62 1220–1227. 10.1093/jnen/62.12.1220 14692698

[B36] Jassam Y. N. Izzy S. Whalen M. Mcgavern D. B. El Khoury J. (2017). Neuroimmunology of traumatic brain injury: time for a paradigm shift. *Neuron* 95 1246–1265. 10.1016/j.neuron.2017.07.010 28910616 PMC5678753

[B37] Jeong H. Kim Y. H. Lee Y. Jung S. J. Oh S. B. (2017). TRPM2 contributes to LPC-induced intracellular Ca2+ influx and microglial cell activation. *Biochem. Biophys. Res. Commun.* 485 301–306. 10.1016/j.bbrc.2017.02.087 28223219

[B38] Ji R. R. Suter M. R. (2007). p38 MAPK, microglial signaling, and neuropathic pain. *Mol. Pain* 3:33. 10.1186/1744-8069-3-33 17974036 PMC2186318

[B39] Jia J. Verma S. Nakayama S. Quillinan N. Grafe M. R. Hurn P. D. (2011). Sex differences in neuroprotection provided by inhibition of TRPM2 channels following experimental stroke. *J. Cereb. Blood Flow Metab.* 31 2160–2168. 10.1038/jcbfm.2011.77 21587268 PMC3210342

[B40] Jiang L. H. Li X. Syed Mortadza S. A. Lovatt M. Yang W. (2018). The TRPM2 channel nexus from oxidative damage to Alzheimer’s pathologies: an emerging novel intervention target for age-related dementia. *Ageing Res. Rev.* 47 67–79. 10.1016/j.arr.2018.07.002 30009973

[B41] Jiang L. H. Yang W. Zou J. Beech D. J. (2010). TRPM2 channel properties, functions and therapeutic potentials. *Expert Opin. Ther. Targets* 14 973–988. 10.1517/14728222.2010.510135 20670202

[B42] Kashio M. Sokabe T. Shintaku K. Uematsu T. Fukuta N. Kobayashi N. (2012). Redox signal-mediated sensitization of transient receptor potential melastatin 2 (TRPM2) to temperature affects macrophage functions. *Proc. Natl. Acad. Sci. U.S.A.* 109 6745–6750. 10.1073/pnas.1114193109 22493272 PMC3340098

[B43] Kashio M. Tominaga M. (2015). Redox signal-mediated enhancement of the temperature sensitivity of transient receptor potential melastatin 2 (TRPM2) elevates glucose-induced insulin secretion from pancreatic islets. *J. Biol. Chem.* 290 12435–12442. 10.1074/jbc.M115.649913 25817999 PMC4424372

[B44] Kettenmann H. Kirchhoff F. Verkhratsky A. (2013). Microglia: new roles for the synaptic stripper. *Neuron* 77 10–18. 10.1016/j.neuron.2012.12.023 23312512

[B45] Kierdorf K. Prinz M. (2017). Microglia in steady state. *J. Clin. Invest.* 127 3201–3209. 10.1172/JCI90602 28714861 PMC5669563

[B46] Knowles H. Li Y. Perraud A. L. (2013). The TRPM2 ion channel, an oxidative stress and metabolic sensor regulating innate immunity and inflammation. *Immunol. Res.* 55 241–248. 10.1007/s12026-012-8373-8 22975787

[B47] Ko S. Y. Wang S. E. Lee H. K. Jo S. Han J. Lee S. H. (2019). Transient receptor potential melastatin 2 governs stress-induced depressive-like behaviors. *Proc. Natl. Acad. Sci. U.S.A.* 116 1770–1775. 10.1073/pnas.1814335116 30642955 PMC6358711

[B48] Kolisek M. Beck A. Fleig A. Penner R. (2005). Cyclic ADP-ribose and hydrogen peroxide synergize with ADP-ribose in the activation of TRPM2 channels. *Mol. Cell* 18 61–69. 10.1016/j.molcel.2005.02.033 15808509

[B49] Kraft R. Grimm C. Grosse K. Hoffmann A. Sauerbruch S. Kettenmann H. (2004). Hydrogen peroxide and ADP-ribose induce TRPM2-mediated calcium influx and cation currents in microglia. *Am. J. Physiol. Cell Physiol.* 286 C129–C137. 10.1152/ajpcell.00331.2003 14512294

[B50] Lee M. Cho T. Jantaratnotai N. Wang Y. T. Mcgeer E. Mcgeer P. L. (2010). Depletion of GSH in glial cells induces neurotoxicity: relevance to aging and degenerative neurological diseases. *FASEB J.* 24 2533–2545. 10.1096/fj.09-149997 20228251

[B51] Li C. Meng L. Li X. Li D. Jiang L. H. (2015). Non-NMDAR neuronal Ca2+-permeable channels in delayed neuronal death and as potential therapeutic targets for ischemic brain damage. *Expert Opin. Ther. Targets* 19 879–892. 10.1517/14728222.2015.1021781 25732672

[B52] Li J. Gao Y. Bao X. Li F. Yao W. Feng Z. (2017). TRPM2: a potential drug target to retard oxidative stress. *Front. Biosci.* 22:1427–1438. 28199210 10.2741/4551

[B53] Li X. Jiang L.-H. (2019). A critical role of the transient receptor potential melastatin 2 channel in a positive feedback mechanism for reactive oxygen species-induced delayed cell death. *J. Cell. Physiol.* 234 3647–3660. 10.1002/jcp.27134 30229906

[B54] Luca A. Calandra C. Luca M. (2018). Molecular bases of Alzheimer’s disease and neurodegeneration: the role of neuroglia. *Aging Dis.* 9 1134–1152. 10.14336/AD.2018.0201 30574424 PMC6284765

[B55] Maiti P. Manna J. Dunbar G. L. (2017). Current understanding of the molecular mechanisms in Parkinson’s disease: targets for potential treatments. *Transl. Neurodegener.* 6:28. 10.1186/s40035-017-0099-z 29090092 PMC5655877

[B56] Marin-Teva J. L. Dusart I. Colin C. Gervais A. Van Rooijen N. Mallat M. (2004). Microglia promote the death of developing Purkinje cells. *Neuron* 41 535–547. 10.1016/S0896-6273(04)00069-8 14980203

[B57] McHugh D. Flemming R. Xu S. Z. Perraud A. L. Beech D. J. (2003). Critical intracellular Ca2+ dependence of transient receptor potential melastatin 2 (TRPM2) cation channel activation. *J. Biol. Chem.* 278 11002–11006. 10.1074/jbc.M210810200 12529379

[B58] Mei Z. Z. Xia R. Beech D. J. Jiang L. H. (2006). Intracellular coiled-coil domain engaged in subunit interaction and assembly of melastatin-related transient receptor potential channel 2. *J. Biol. Chem.* 281 38748–38756. 10.1074/jbc.M607591200 17060318 PMC1698503

[B59] Meister A. Anderson M. E. (1983). Glutathione. *Annu. Rev. Biochem.* 52 711–760. 10.1146/annurev.bi.52.070183.003431 6137189

[B60] Menassa D. A. Gomez-Nicola D. (2018). Microglial dynamics during human brain development. *Front. Immunol.* 9:1014. 10.3389/fimmu.2018.01014 29881376 PMC5976733

[B61] Miller B. A. Zhang W. (2011). TRP channels as mediators of oxidative stress. *Adv. Exp. Med. Biol.* 704 531–544. 10.1007/978-94-007-0265-3_29 21290315

[B62] Miyake T. Shirakawa H. Kusano A. Sakimoto S. Konno M. Nakagawa T. (2014). TRPM2 contributes to LPS/IFNγ-induced production of nitric oxide via the p38/JNK pathway in microglia. *Biochem. Biophys. Res. Commun.* 444 212–217. 10.1016/j.bbrc.2014.01.022 24462864

[B63] Miyanohara J. Kakae M. Nagayasu K. Nakagawa T. Mori Y. Arai K. (2018). TRPM2 channel aggravates CNS inflammation and cognitive impairment via activation of microglia in chronic cerebral hypoperfusion. *J. Neurosci.* 38 3520–3533. 10.1523/JNEUROSCI.2451-17.2018 29507145 PMC6596050

[B64] Ostapchenko V. G. Chen M. Guzman M. S. Xie Y. F. Lavine N. Fan J. (2015). The transient receptor potential melastatin 2 (TRPM2) channel contributes to β-amyloid oligomer-related neurotoxicity and memory impairment. *J. Neurosci.* 35 15157–15169. 10.1523/JNEUROSCI.4081-14.2015 26558786 PMC6605355

[B65] Perraud A. L. Fleig A. Dunn C. A. Bagley L. A. Launay P. Schmitz C. (2001). ADP-ribose gating of the calcium-permeable LTRPC2 channel revealed by Nudix motif homology. *Nature* 411 595–599. 10.1038/35079100 11385575

[B66] Perraud A. L. Takanishi C. L. Shen B. Kang S. Smith M. K. Schmitz C. (2005). Accumulation of free ADP-ribose from mitochondria mediates oxidative stress-induced gating of TRPM2 cation channels. *J. Biol. Chem.* 280 6138–6148. 10.1074/jbc.M411446200 15561722

[B67] Ramirez A. I. De Hoz R. Salobrar-Garcia E. Salazar J. J. Rojas B. Ajoy D. (2017). The role of microglia in retinal neurodegeneration: Alzheimer’s Disease, Parkinson, and glaucoma. *Front. Aging Neurosci.* 9:214 10.3389/fnagi.2017.00214 PMC5498525 28729832

[B68] Ru X. Yao X. (2014). TRPM2: a multifunctional ion channel for oxidative stress sensing. *Sheng Li Xue Bao* 66 7–15. 24553864

[B69] Saijo K. Glass C. K. (2011). Microglial cell origin and phenotypes in health and disease. *Nat. Rev. Immunol.* 11 775–787. 10.1038/nri3086 22025055

[B70] Salter M. W. Stevens B. (2017). Microglia emerge as central players in brain disease. *Nat. Med.* 23 1018–1027. 10.1038/nm.4397 28886007

[B71] Sano Y. Inamura K. Miyake A. Mochizuki S. Yokoi H. Matsushime H. (2001). Immunocyte Ca2+ influx system mediated by LTRPC2. *Science* 293 1327–1330. 10.1126/science.1062473 11509734

[B72] Schilling T. Lehmann F. Ruckert B. Eder C. (2004). Physiological mechanisms of lysophosphatidylcholine-induced de-ramification of murine microglia. *J. Physiol.* 557 105–120. 10.1113/jphysiol.2004.060632 15020687 PMC1665039

[B73] Sheikh A. M. Nagai A. Ryu J. K. Mclarnon J. G. Kim S. U. Masuda J. (2009). Lysophosphatidylcholine induces glial cell activation: role of rho kinase. *Glia* 57 898–907. 10.1002/glia.20815 19115379

[B74] Shetty A. K. Kodali M. Upadhya R. Madhu L. N. (2018). Emerging anti-aging strategies - scientific basis and efficacy. *Aging Dis.* 9 1165–1184. 10.14336/AD.2018.1026 30574426 PMC6284760

[B75] Shimizu T. Dietz R. M. Cruz-Torres I. Strnad F. Garske A. K. Moreno M. (2016). Extended therapeutic window of a novel peptide inhibitor of TRPM2 channels following focal cerebral ischemia. *Exp. Neurol.* 283 151–156. 10.1016/j.expneurol.2016.06.015 27317297 PMC5240152

[B76] Shimizu T. Macey T. A. Quillinan N. Klawitter J. Perraud A. L. Traystman R. J. (2013). Androgen and PARP-1 regulation of TRPM2 channels after ischemic injury. *J. Cereb. Blood Flow Metab.* 33 1549–1555. 10.1038/jcbfm.2013.105 23801245 PMC3790922

[B77] Sierra A. Encinas J. M. Deudero J. J. Chancey J. H. Enikolopov G. Overstreet-Wadiche L. S. (2010). Microglia shape adult hippocampal neurogenesis through apoptosis-coupled phagocytosis. *Cell Stem Cell* 7 483–495. 10.1016/j.stem.2010.08.014 20887954 PMC4008496

[B78] Sohal R. S. Weindruch R. (1996). Oxidative stress, caloric restriction, and aging. *Science* 273 59–63. 10.1126/science.273.5271.59 8658196 PMC2987625

[B79] Song K. Wang H. Kamm G. B. Pohle J. Reis F. C. Heppenstall P. (2016). The TRPM2 channel is a hypothalamic heat sensor that limits fever and can drive hypothermia. *Science* 353 1393–1398. 10.1126/science.aaf7537 27562954 PMC7612276

[B80] Song L. Pei L. Yao S. Wu Y. Shang Y. (2017). NLRP3 inflammasome in neurological diseases, from functions to therapies. *Front. Cell Neurosci.* 11:63 10.3389/fncel.2017.00063 PMC5343070 28337127

[B81] Sousa C. Golebiewska A. Poovathingal S. K. Kaoma T. Pires-Afonso Y. Martina S. (2018). Single-cell transcriptomics reveals distinct inflammation-induced microglia signatures. *EMBO Rep.* 19:e46171. 10.15252/embr.201846171 30206190 PMC6216255

[B82] Sun Y. Sukumaran P. Selvaraj S. Cilz N. I. Schaar A. Lei S. (2018). TRPM2 promotes neurotoxin MPP+/MPTP-induced cell death. *Mol. Neurobiol.* 55 409–420. 10.1007/s12035-016-0338-9 27957685 PMC5468501

[B83] Syed Mortadza S. A. Sim J. A. Neubrand V. E. Jiang L. H. (2018). A critical role of TRPM2 channel in Aβ42 -induced microglial cell activation and generation of tumor necrosis factor-α. *Glia* 66 562–575. 10.1002/glia.23265 29143372

[B84] Syed Mortadza S. A. Sim J. A. Stacey M. Jiang L. H. (2017). Signalling mechanisms mediating Zn2+-induced TRPM2 channel activation and cell death in microglial cells. *Sci. Rep.* 7:45032. 10.1038/srep45032 28322340 PMC5359577

[B85] Syed Mortadza S. A. Wang L. Li D. Jiang L. H. (2015). TRPM2 channel-mediated ROS sensitive Ca2+ signaling mechanisms in immune cells. *Front. Immunol.* 6:407. 10.3389/fimmu.2015.00407 26300888 PMC4528159

[B86] Szepesi Z. Manouchehrian O. Bachiller S. Deierborg T. (2018). Bidirectional microglia-neuron communication in health and disease. *Front. Cell Neurosci.* 12:323. 10.3389/fncel.2018.00323 30319362 PMC6170615

[B87] Takahashi N. Kozai D. Kobayashi R. Ebert M. Mori Y. (2011). Roles of TRPM2 in oxidative stress. *Cell Calcium* 50 279–287. 10.1016/j.ceca.2011.04.006 21616534

[B88] Tan C. H. McNaughton P. A. (2016). The TRPM2 ion channel is required for sensitivity to warmth. *Nature* 536 460–463. 10.1038/nature19074 27533035 PMC5720344

[B89] Tay T. L. Savage J. C. Hui C. W. Bisht K. Tremblay M. E. (2017). Microglia across the lifespan: from origin to function in brain development, plasticity and cognition. *J. Physiol.* 595 1929–1945. 10.1113/JP272134 27104646 PMC5350449

[B90] Togashi K. Hara Y. Tominaga T. Higashi T. Konishi Y. Mori Y. (2006). TRPM2 activation by cyclic ADP-ribose at body temperature is involved in insulin secretion. *EMBO J.* 25 1804–1815. 10.1038/sj.emboj.7601083 16601673 PMC1456947

[B91] Tong Q. Zhang W. Conrad K. Mostoller K. Cheung J. Y. Peterson B. Z. (2006). Regulation of the transient receptor potential channel TRPM2 by the Ca2+ sensor calmodulin. *J. Biol. Chem.* 281 9076–9085. 10.1074/jbc.M510422200 16461353

[B92] Tóth B. Csanády L. (2010). Identification of direct and indirect effectors of the transient receptor potential melastatin 2 (TRPM2) cation channel. *J. Biol. Chem.* 285 30091–30102. 10.1074/jbc.M109.066464 20650899 PMC2943302

[B93] Toth B. Iordanov I. Csanady L. (2015). Ruling out pyridine dinucleotides as true TRPM2 channel activators reveals novel direct agonist ADP-ribose-2’-phosphate. *J. Gen. Physiol.* 145 419–430. 10.1085/jgp.201511377 25918360 PMC4411260

[B94] Tschopp J. Schroder K. (2010). NLRP3 inflammasome activation: the convergence of multiple signalling pathways on ROS production? *Nat. Rev. Immunol.* 10 210–215. 10.1038/nri2725 20168318

[B95] Tsuda M. Masuda T. Tozaki-Saitoh H. Inoue K. (2013). Microglial regulation of neuropathic pain. *J. Pharmacol. Sci.* 121 89–94. 10.1254/jphs.12R14CP 23337437

[B96] Tsutsui M. Hirase R. Miyamura S. Nagayasu K. Nakagawa T. Mori Y. (2018). TRPM2 exacerbates central nervous system inflammation in experimental autoimmune encephalomyelitis by increasing production of CXCL2 chemokines. *J. Neurosci.* 38 8484–8495. 10.1523/JNEUROSCI.2203-17.2018 30201769 PMC6596171

[B97] Voet S. Prinz M. Van Loo G. (2018). Microglia in central nervous system inflammation and multiple sclerosis pathology. *Trends Mol. Med.* 25 112–123. 10.1016/j.molmed.2018.11.005 30578090

[B98] Wang L. Fu T. M. Zhou Y. Xia S. Greka A. Wu H. (2018). Structures and gating mechanism of human TRPM2. *Science* 362:eaav4809. 10.1126/science.aav4809 30467180 PMC6459600

[B99] Wang W. Y. Tan M. S. Yu J. T. Tan L. (2015). Role of pro-inflammatory cytokines released from microglia in Alzheimer’s disease. *Ann. Transl. Med.* 3:136. 10.3978/j.issn.2305-5839.2015.03.49 26207229 PMC4486922

[B100] Wei L. Syed Mortadza S. A. Jiang L. H. (2018). Melastatin-related transient receptor potential 2 channel in Abeta42-induced neuroinflammation: implications to Alzheimer’s disease mechanism and development of therapeutics. *Neural Regen. Res.* 13 419–420. 10.4103/1673-5374.228720 29623922 PMC5900500

[B101] White C. S. Lawrence C. B. Brough D. Rivers-Auty J. (2017). Inflammasomes as therapeutic targets for Alzheimer’s disease. *Brain Pathol.* 27 223–234. 10.1111/bpa.12478 28009077 PMC8029266

[B102] Xia R. Mei Z. Z. Mao H. J. Yang W. Dong L. Bradley H. (2008). Identification of pore residues engaged in determining divalent cationic permeation in transient receptor potential melastatin subtype channel 2. *J. Biol. Chem.* 283 27426–27432. 10.1074/jbc.M801049200 18687688 PMC2562080

[B103] Xu C. Macciardi F. Li P. P. Yoon I. S. Cooke R. G. Hughes B. (2006). Association of the putative susceptibility gene, transient receptor potential protein melastatin type 2, with bipolar disorder. *Am. J. Med. Genet. B Neuropsychiatr. Genet.* 141B, 36–43. 10.1002/ajmg.b.30239 16252251

[B104] Yamamoto S. Shimizu S. (2016). Targeting TRPM2 in ROS-coupled diseases. *Pharmaceuticals* 9:E57. 10.3390/ph9030057 27618067 PMC5039510

[B105] Ye M. Yang W. Ainscough J. F. Hu X. P. Li X. Sedo A. (2014). TRPM2 channel deficiency prevents delayed cytosolic Zn2+ accumulation and CA1 pyramidal neuronal death after transient global ischemia. *Cell Death Dis.* 5:e1541 10.1038/cddis.2014.494 PMC4260752 25429618

[B106] Yirmiya R. Rimmerman N. Reshef R. (2015). Depression as a microglial disease. *Trends Neurosci.* 38 637–658. 10.1016/j.tins.2015.08.001 26442697

[B107] Yürüker V. Naziroðlu M. §cenol N. (2015). Reduction in traumatic brain injury-induced oxidative stress, apoptosis, and calcium entry in rat hippocampus by melatonin: possible involvement of TRPM2 channels. *Metab. Brain Dis.* 30 223–231. 10.1007/s11011-014-9623-3 25339252

[B108] Zhang W. Chu X. Tong Q. Cheung J. Y. Conrad K. Masker K. (2003). A novel TRPM2 isoform inhibits calcium influx and susceptibility to cell death. *J. Biol. Chem.* 278 16222–16229. 10.1074/jbc.M300298200 12594222

[B109] Zhang Z. Tóth B. Szollosi A. Chen J. Csanády L. (2018). Structure of a TRPM2 channel in complex with Ca2+ explains unique gating regulation. *eLife* 7:e36409. 10.7554/eLife.36409 29745897 PMC5976436

[B110] Zhong J. Amina S. Liang M. Akther S. Yuhi T. Nishimura T. (2016). Cyclic ADP-ribose and heat tegulate oxytocin release via CD38 and TRPM2 in the hypothalamus during social or psychological stress in mice. *Front. Neurosci.* 10:304 10.3389/fnins.2016.00304 PMC4956647 27499729

